# Effects of resilience, social support, and academic self-efficacy, on mental health among Peruvian university students during the pandemic: the mediating role of digital inclusion

**DOI:** 10.3389/fpsyg.2024.1282281

**Published:** 2024-07-08

**Authors:** Mónica Cassaretto, Agustín Espinosa, Cecilia Chau

**Affiliations:** Department of Psychology, Pontificia Universidad Católica del Perú, Lima, Peru

**Keywords:** mental health, resilience, academic self-efficacy, social support, digital inclusion, COVID-19, university students

## Abstract

**Background:**

Mental health of university students has been impacted during the pandemic, highlighting the importance of understanding its psychosocial determinants. Nevertheless, there has been limited exploration into whether the digital inclusion conditions for remote education could mediate the effects that variables such as resilience, social support, and academic self-efficacy may have on mental health. Considering the above, there is evidence that shows a consistent relationship between resilience, social support and academic self-efficacy on mental health, to the extent that these are psychological variables. On the other hand, digital inclusion, which comprehends a contextual variable, not a psychological one, related to ICT access opportunities and mainly focused on the quality of Internet access, should be analyzed in a differential manner.

**Objectives:**

This study seeks to analyze the effect of resilience, social support and academic self-efficacy, on the mental health of a group of Peruvian university students; in addition, it seeks to analyze the mediating role of digital inclusion.

**Materials and methods:**

A cross-sectional study was conducted with 3,147 undergraduate students from a private university in Lima, Perú. From August to October 2020, data were collected online through questionnaire, this include The Depression, Anxiety and Stress Scale 21 (DASS-21), The 10-item version of the Connor-Davidson Resilience Scale (CD-RISC10), The Multidimensional Scale of Perceived Social Support (EMASP), The Perceived Self-Efficacy Specific for Academic Situations Scale (EAPESA) and to measure digital inclusion, the Perceived Quality of Internet Access reported by the students. The levels of participants’ anxiety, depression and stress were described using frequency and percentage. Pearson Correlation test was used to measure the correlation between the variables and a Path analysis was conducted. Finally, The PROCESS macro for SPSS (Model 4) was applied to examine the mediating effect of the model controlling gender variable.

**Results:**

The results revealed significant levels of extremely severe symptoms of anxiety (36.8%), depression (33.4%) and stress (18.1%) among the participants. A path analysis, which indicated that resilience (*β* = −0.346), social support (*β* = −0.189), academic self-efficacy (*β* = −0.060) and digital inclusion (*β* = −0.089) had significant impact on students’ General Distress. In addition, digital inclusion plays a partial mediation role with low but significant effect size in the relationship between resilience, social support and self-efficacy with mental health.

**Conclusion:**

Mental health of university students during the pandemic shows alarming levels of general or emotional distress. The findings indicate that resilience, social support and self-efficacy protect college students’ mental health by reducing general distress. However, the study shows that when there is a digital divide around internet quality the impact of these factors is affected.

## 1 Introduction

### 1.1 Mental health in university students in the COVID-19 pandemic

The COVID-19 pandemic has generated significant repercussions in the health status of people around the world. This context has brought with it a notable decrease in people’s well-being and quality of life [Organización Panamericana de la Salud (OPS), 2021a]. Although mental health is defined as a state of mental well-being that enables people to cope with the stresses of life, realize their abilities, learn well, work well, and contribute to their community [World Health Organization (WHO), 2022]; most research in this field has focused predominantly on the recognition of emotional or general distress signs. Consequently, numerous studies have highlighted a high prevalence of mental health problems among university students, characterized especially by symptoms of anxiety, depression, and stress (Cao et al., 2020; Debowska et al., 2020; Wang et al., 2020; Wathelet et al., 2020; Li et al., 2021).

In the university population, in addition to the irremediable effects of the pandemic, such as altered lifestyles, economic difficulties, unemployment, loss of loved ones, and fear of illness, there was also the forced transition to virtual education (Sahu, 2020; Xie et al., 2020; Tasso et al., 2021). This, because the pandemic generated changes in education policies, and in 2020 all face-to-face academic activities were ordered to cease in more than 190 countries, with the aim of protecting the health of university students [Ali, 2020; Comisión Económica para América Latina (CEPAL), 2020].

### 1.2 Distance learning in higher education, digital inclusion, and mental health during COVID-19 pandemic

The Peruvian higher education system underwent significant disruption due to the pandemic, which forced an abrupt shift to distance education (Vilela et al., 2021). In this scenario, professors had to adapt by redesigning their content and mastering new virtual platforms. Consequently, many professors required intensive training in information and communication technologies (ICTs). However, this adjustment proved to be challenging, leading to reported difficulties in managing virtual platforms, experiencing connectivity problems, and grappling with some digital skills deficiencies (Huanca-Arohuanca et al., 2020; Lovon and Cisneros, 2020). Additionally, striking the right balance in using the tools offered by virtual platforms posed a learning curve for many teachers. As a result, numerous students reported being overwhelmed by virtual tasks and activities during the initial semesters of the pandemic (Lovon and Cisneros, 2020; Vilela et al., 2021).

This process has highlighted the significant digital gap that exists in Peru. Private universities and those located in the capital city have had major advantages in accessing virtual education (Vilela et al., 2021). The disadvantages resulting from the lack of technological resources and infrastructure communication system contributed to the frustration of the students, who were unable to fully engage in their academic activities. This, combined with academic overload and the pressures of the pandemic context, may have impacted students’ mental health, potentially explaining the 15% dropout rate reported during the first year of the pandemic (Lovon and Cisneros, 2020; Saravia and Palomino, 2022; Leitón et al., 2023).

In this context, digital inclusion is defined as the search for equal access to information and communications technologies (ICTs), so that all people have the same opportunities to make appropriate use of them (Selwyn and Facer, 2007; Seale, 2009). Historically, digital inclusion came from the concept “digital divide” proposed by Foster and Burkowsi (2007), which refers to the social differences between people with and without access to the Internet. In summary, digital inclusion involves various elements, such as the absence of access to electronic devices, inadequate internet connectivity and quality, and a lack of knowledge in device usage. Its importance has heightened due to the immediate effects of the COVID-19 pandemic, notably in education, where digital tools like cell phones, computers, and laptops were utilized for academic purposes (Ali, 2020; Maqableh and Alia, 2021). Virtual education, while effective in controlling COVID 19 transmission and revolutionizing teaching methods, information access, and educational content creation, has also unveiled the pre-existing inequality in the availability of such services [Comisión Económica para América Latina (CEPAL), 2020].

The digital gap is a persistent issue in education in Peru and other regional countries, especially affecting vulnerable sectors lacking quality devices and Internet access for effective online learning (Ministerio de Educación [Minedu], 2022). Globally, university students to be more proficient with virtual softwares and platforms (Zhao et al., 2021). However, significant gaps in digital inclusion exist based on socioeconomic and territorial factors (Zhao et al., 2021). During the pandemic, many university students incurred extra costs to secure quality technology. Internet service contracts were adjusted to improve connectivity (Saravia and Palomino, 2022). Additionally, certain universities, recognizing the digital gap, initiated measures like the *Connectivity Fund*, providing modems and electronic devices to disadvantaged students (Lovon and Cisneros, 2020).

Recent studies have found that inequality in access to quality digital services compromises not only the student’s possibility to receive a better education but also impacts their sense of belonging, motivation to study, academic performance and mental health (Xie et al., 2020; Maqableh and Alia, 2021). The sudden shift toward virtual education in the case of university students would be found to be related to a decrease in satisfaction with the new learning modality (Coman et al., 2020; Tejedor et al., 2020; Maqableh and Alia, 2021; Chanto and Loáiciga, 2022) and an increase in the risk of developing symptoms of depression, anxiety and stress (Alemany-Arrebola et al., 2020; Lamanauskas and Makarskaite-Petkeviciene, 2021). In addition, it has been found that the quality of internet service and computer access would have a relationship with students’ psychological distress (Fawaz and Samaha, 2021). Several studies have investigated how university students have adapted to virtual education. Factors such as increased academic workload, lack of clear guidelines on the new teaching method, concerns about the quality of learning and the instability of Internet connections have collectively led to lower acceptance of this new teaching modality (Coman et al., 2020; Tejedor et al., 2020; Debeş, 2021; Chanto and Loáiciga, 2022).

### 1.3 Psychosocial predictors of mental health during COVID-19 pandemic

Several investigations over time indicate that certain variables such as resilience, academic self-efficacy and social support play a fundamental role in mental health, as they interact in a complex manner to influence the ability of individuals to face and adapt to challenges in various contexts and situations (Zhou and Yu, 2021; Noh and Park, 2022; Ortiz-Calvo et al., 2022; Warshawski, 2022). Specifically, resilience has been found to have a significant impact on mental health, as it strengthens people’s ability to overcome adversity, thus promoting greater well-being and a better quality of life (Burns and Anstey, 2010; Lee et al., 2013). In the pandemic context, resilience has been an important factor for mental health, as it allowed people to cope with the multiple stressors and challenges associated with the health crisis, strengthening their adaptive capacities (Calle and Urigüen, 2022; Ortiz-Calvo et al., 2022; Valero et al., 2022). In university students, positive associations have been found between resilience and positive affect, life satisfaction and psychological well-being (Noh and Park, 2022; Nuñez and Vásquez, 2022).

Likewise, social support plays a critical role in mental health as having support from significant others strengthens psychological well-being, reduces mental health problems, provides a sense of belonging and meaningful connections (Rueger et al., 2016; Uchino et al., 2016; Alsubaie et al., 2019; Hu et al., 2020). During the pandemic, emotional and practical support from the social network enabled the preservation of well-being during these difficult times (Goselin and Rickert, 2022; Noh and Park, 2022; Ortiz-Calvo et al., 2022). In college students, it has been found that having social support from peers allows coping with academic stress and uncertainty, thus contributing to improve student’s psychological well-being and reduce mental health issues such as anxiety, depression, and stress (Alsubaie et al., 2019; Goselin and Rickert, 2022; Noh and Park, 2022).

Finally, academic self-efficacy, also exerts a significant effect on mental health, since the belief in one’s own ability to face challenges at the academic level not only improves academic performance, but also contributes to reduce stress, anxiety and promote greater psychological well-being in students (Domínguez-Lara, 2014; Ampuero et al., 2022; Andersson et al., 2022). However, the pandemic-induced uncertainty and alterations in the teaching-learning landscape have resulted in reduced academic self-efficacy among university students. This decline has led to a decreased willingness to tackle demanding tasks, self-monitor their learning, seek assistance from peers or professors, and proactively pursue actions that facilitate their successful adjustment to their academic pursuits (Navarro-Mateu et al., 2020). The decrease in academic self-efficacy perceived by students has been associated with clinical indicators of anxiety, depression and stress (Alemany-Arrebola et al., 2020; Navarro-Mateu et al., 2020; Ampuero et al., 2022; Carranza et al., 2022; Ortiz-Calvo et al., 2022). In addition, recent studies find a positive relationship between academic self-efficacy, motivation, academic performance and satisfaction with studies in university students (She et al., 2021; Talsma et al., 2021; Carranza et al., 2022).

The dynamics between academic self-efficacy, social support, and resilience in the mental health domain are complex and multifaceted, as it is found that the perceived support of a significant other (social support), and the individual’s capacity to overcome adversity (resilience), are directly related to students’ academic self-efficacy beliefs (Zhou and Yu, 2021). In addition, studies find that social support and resilience are inversely associated with mental health issues such as anxiety, depression, and stress (Alsubaie et al., 2019; Goselin and Rickert, 2022; Noh and Park, 2022). Noteworthy are the positive associations between resilience and social support with protective mental health factors such as psychological well-being, perceived social support, life satisfaction, and positive affect (Burns and Anstey, 2010; Lee et al., 2013; Rueger et al., 2016; Hu et al., 2020).

While the pandemic may have subsided, it remains crucial to underscore the lasting impact on students’ mental health. University life exposes students to a multitude of demands and challenges across different domains, often leading to heightened distress among young individuals. The aftermath of the Covid-19 pandemic has exacerbated some of these stressors in recent years, causing a broader decline in overall health; therefore, it is important to study the variables that predict mental health, with special emphasis on psychological variables, since they are of utmost importance to implement educational and institutional policies and reforms. Therefore, the main objective of the present study is to analyze the effect of resilience, social support and academic self-efficacy, on the mental health of a group of Peruvian university students.

Considering the above, there is evidence that shows a consistent relationship between self-efficacy, social support and resilience on mental health, to the extent that these are psychological variables. On the other hand, digital inclusion, which comprehends a contextual variable, not a psychological one, related to ICT access opportunities and mainly focused on the quality of Internet access, should be analyzed in a differential manner. Thus, specifically, the aim is to analyze the mediating role of digital inclusion on the effect of self-efficacy, social support and academic resilience on the mental health of the participants. In the present study, mental health will be measured, in its negative connotation, through an indicator of general distress. In this sense, it is hypothesized that resilience, social support and academic self-efficacy will have a significant and negative effect on general distress. Likewise, the effect sizes of these relationships are expected to increase when greater perceived digital inclusion is introduced as a mediating variable.

## 2 Materials and methods

### 2.1 Participants

The present study was initiated at the request of university authorities. The sampling approach utilized was non-probabilistic convenience sampling, based on students’ interest, availability, and accessibility to complete an online questionnaire. A total of 3,147 undergraduate students from a private university in Lima, Peru responded to the survey. The majority of the participants were female (62.6%), followed by males (36.4%), while a smaller percentage (1%) preferred not to answer the question. Participants ranged in age from 18 to 65 years (*M* = 21.02; *SD* = 3.38). A slightly more than half of the sample mentioned studying a degree in Faculties of Arts and Humanities, Social Sciences, and Law (55%), followed by a degree in Faculty of STEM (33.3%) while a much smaller number in Fine Arts (7.7%).

### 2.2 Instruments

#### 2.2.1 Mental health

The Depression, Anxiety and Stress Scale 21 (DASS-21, Lovibond and Lovibond, 1995), in its Spanish version by Daza et al. (2002) was used. The DASS-21 measures negative emotional states by means of 21 items grouped into three factors: depressive symptoms, symptoms of anxiety, and symptoms of stress, each consisting of 7 items (e.g., “I could not seem to experience any positive feeling at all,” “I felt scared without any good reason,” “I found it difficult to relax”). It also evaluates a general component called General or Emotional Distress, which when interpreted inversely gives an indicator of mental health, so that the higher the score on the general scale, the higher the general distress and lower the mental health. This scale has a 4-point Likert response format (0 = *It has not happened to me*; 3 = *It has happened to me a lot, or most of the time*). In the present study the internal consistency of the test obtained an alpha of 0.95 and between 0.85 to 0.91 for the specific scales.

#### 2.2.2 Resilience

The 10-item version of the Connor-Davidson Resilience Scale (CD-RISC10; Connor and Davidson, 2003) was used. This scale assesses different aspects of resilience such as flexibility, sense of self-efficacy, ability to regulate emotions, optimism and cognitive approach under stress. This instrument has a Likert-type response format with options from 0 (*never*) to 4 (*almost always*). It consists of 10 statements that assess resilience (e.g., “I can deal with whatever comes”). In the current study, the Spanish version of Notario-Pacheco et al. (2011), who validated the instrument in 681 Spanish university students, was used. Cronbach’s alpha coefficient was used for reliability, finding an alpha of 0.85. In the present study, the test presented an internal consistency alpha of 0.92.

#### 2.2.3 Social support

The Multidimensional Scale of Perceived Social Support (EMASP, Zimet et al., 1988) was used. This scale evaluates the levels of social support that individuals perceive in relation to three sources: family, friends and relevant people; the sum of these three aspects makes it possible to obtain a global assessment of the perception of support that the person possesses. The Spanish adaptation of Landeta and Calvete (2002) was used; this test has 12 items (e.g., “There is a special person with whom I can share joys and sorrows”) that are answered in a Likert format with 6 possible response alternatives, where 1 is equivalent to “*completely disagree*” and 6 to “*completely agree*.” Regarding the reliability of this test, it has an alpha coefficient of 0.89. In the present study the test has a reliability of 0.91.

#### 2.2.4 Academic self-efficacy

The Specific Perceived Self-Efficacy Scale for Academic Situations (EAPESA) created in Peru by Domínguez-Lara (2014) was used. It measures the students’ assessment of their capabilities for the development and accomplishment of activities specific to the educational environment. It is a nine-item instrument constructed and validated with a sample of 448 Peruvian university students (e.g. “I feel confident to deal with situations that test my academic ability”). The test has a Likert response format with scores ranging from 1 = *never* to 4 = *always*. Regarding reliability, Cronbach’s alpha coefficient is adequate (*α* = 0.88). In this study the internal consistency alpha index of the test is 0.91.

#### 2.2.5 Digital inclusion

As previously established, digital inclusion comprises a set of factors that allow people to access ICTs. The digital inclusion variables controlled in the present research included the following (1) access to digital devices, (2) personal skills (knowledge) for the use of digital devices, and (3) perceived quality of Internet connection. However, in the case of the present research, 98.5% had easy access to devices to carry out their studies and had basic digital competencies. Therefore, the digital inclusion measure focused on the perceived quality of Internet access based on a simple question created for this study (*How would you rate your Internet connection?*) answered in a Likert format with 5 options, ranging from 1 = *Very bad,* 2 = *bad*, 3 = *fair*, 4 = *good* and 5 = *Very good*.

### 2.3 Procedure

The present investigation is part of an institutional project promoted by the Consortium of Universities (association of 4 private universities in the city of Lima), with the main objective of attending and responding to the mental health needs of university students during the pandemic context for the COVID-19. Specifically, the results reported in the present investigation correspond to a sample selected in one of the universities belonging to the consortium.

The data collection was conducted in the period from August to October 2020 during the pandemic context. The inclusion criteria: the student must be of age and enrolled in courses at the university during the semester 2020–1. University students were invited to participate through the institutional e-mail. Those students who met the inclusion criteria and gave their consent to participate were presented with an online survey comprising a data sheet and questionnaires on the virtual platform Google Forms.

As part of the ethical considerations, before answering the questionnaire, the study participants were presented with the terms of the informed consent, where they were informed of the purpose of the research, as well as the use that would be made of the information collected from their responses. Likewise, participants were informed that their participation was completely voluntary, anonymous and that they could withdraw from the research at any time without any harm being done to them. At the conclusion of their participation in the survey, the students received information on the university’s wellness offices, their contact information and the services they provided.

### 2.4 Statistical analysis

In order to achieve the objectives of the study, several statistical techniques were used. First, the prevalence of indicators of General Distress (inverse measure of mental health) in the sample of students was analyzed at a descriptive level. Secondly, mean comparison analyses were conducted on the indicators of General Distress by sex (male versus female) of the participants. In the third step, the associations between all the variables included in the study were analyzed by means of Pearson’s correlation analyses. In a fourth step, responding to the general objective of the study, a Path analysis was conducted, where resilience, social support, academic self-efficacy as predictor variables, digital inclusion both as predictor and mediating variables and sex as control variable, since they were considered relevant in the explanation of general distress. Finally, to respond to the specific objective of the study, in a fifth step, a complementary simple mediation analyses were performed with Hayes’ macro model 4, to estimate accurately the mediating role of digital inclusion (mediating variable) on the effect of the psychological indicators of resilience, social support and academic self-efficacy (predictor variables or covariates, depending on the analysis) on General Distress (dependent variable) was analyzed. Due the effect of gender on general distress, this variable was introduced as control measure in the mediation analysis. At the latter stage, the bootstrapping statistical inference method was also processed in each mediation analysis performed with 5,000 samples to estimate the significance of the mediating effect of digital inclusion.

## 3 Results

At a descriptive level, the results show that there is a high prevalence of mental health affectation during the pandemic context in the student population investigated. Thus, severe and extremely severe levels of General Distress dimensions comprise 47.7% of anxiety, 47.8% depression and 41.5% stress in the total sample. Considering severe or extremely severe expressions of General Distress as a whole, this reached 52.6% of the total sample, with 17.2% of the participants presenting only one of the distress patterns, 16.4% presenting two, and 29% presenting all three distress patterns evaluated at levels considered critical (Table 1).

**Table 1:** Prevalence of indicators of general distress in the sample according to levels of intensity.

Intensity levels	Anxiety	Depression	Stress
Absence	26.8%	21.3%	28.7%
Mild	6.6%	10.4%	11.9%
Moderated	19.0%	20.5%	17.9%
Severe	10.9%	14.4%	23.4%
Extremely severe	36.8%	33.4%	18.1%
Total	100%	100%	100%

*N* = 3,147.

Relating the prevalence of General Distress by gender, it can be seen that, on average, it is significantly lower in men (*M* = 26.87, *SD* = 15.52) than in women (*M* = 30.37, *SD* = 15.584, *t*(3115) = −6.056, *p < 0*.001, Mean Difference = −3.5, 95%CI of Mean Difference [−4.63, −2.37]).

Regarding the factors associated with General Distress, several Pearson correlation analyses were processed, which show that, consistently with expectations, General Distress is inversely associated, with medium to large effect sizes (see Richard et al., 2003), with resilience, social support and academic self-efficacy. The study found that there is a significant and moderate-sized inverse relationship between General Distress and the indicator of digital inclusion (perceived quality of the Internet connection). The correlation matrix between the variables referred to is presented below (Table 2).

**Table 2:** Pearson’s correlation analysis between study variables.

		1.	2.	3.	4.	5.
1.	General distress	-	−0.503**	−0.398**	−0.390**	−0.213**
2.	Resilience		-	0.488**	0.646**	0.203**
3.	Social support			-	0.399**	0.214**
4.	Academic self-efficacy				-	0.198**
5.	Digital inclusion					-

**p < 0.001.

To analyze the joint effect of resilience, social support and self-efficacy as predictor variables, as well as, digital inclusion as predictor and mediating variable, a path analysis was processed, which yielded one model with good fitness (χ^2^/df = 0.101, *CFI* = 0.99, *RMSEA* = 0.0001 *CI90* [0.0001–0.032]). The model shows that resilience (*β* = −0.346) appears as the component with the major effect on General Distress, followed by global social support (*β* = −0.189), digital inclusion (*β* = −0.089) and self-efficacy (*β* = −0.060), respectively. Additionally, digital inclusion appears as partially mediating the relations between resilience, global support and academic self-efficacy on General Distress.

**Figure 1:**
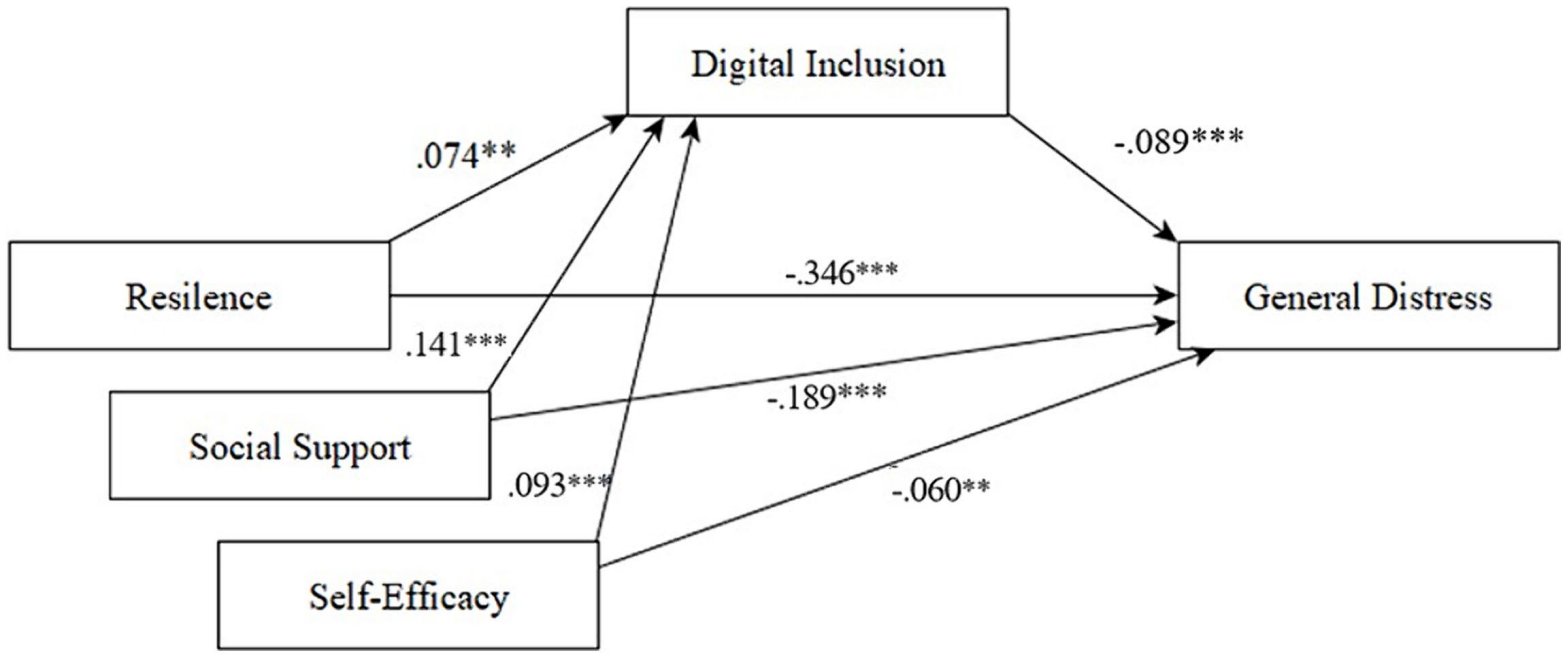
Mediation model with digital inclusion as a mediator between study variables and general distress (Gender variable as control omitted in the graph).

This study emphasizes the importance of General Distress and its relations to factors such as resilience, social support, and academic self-efficacy, aligning with previous research. However, the perceived quality of internet connection, a digital inclusion measure, has emerged as a predictor of General Distress. Yet, it is distinct conceptually from the aforementioned psychological factors. In essence, digital inclusion operates on a separate analytical level. To explore this further, the study employs three simple mediation analyses. The goal is to determine whether digital inclusion acts as a mediator of the impact of the psychological constructs (resilience, social support, and academic self-efficacy) on General Distress. Thus, Figure 1 shows a summary of the models analyzed with the Hayes macro, together with a bootstrapping analysis with 5,000 samples. This summary is possible given that the three models are mathematically equivalent (Hayes, 2022).

Table 3 shows the indirect and total effects of each study variable on General Distress, with digital inclusion as the mediator. The results of the mediation analysis indicate that digital inclusion partially mediates the relationship between the study variables and General Distress.

**Table 3:** Total and indirect effect of study variables on general distress.

	Indirect effect	Total effect
Resilience	−0.007 [−0.012; −0.002]	−0.353***
Social support	−0.013 [−0.018; −0.008]	−0.201***
Academic self-efficacy	−0.082 [−0.014; −0.004]	−0.070***

The values inside the brackets represent the upper and lower limits of the 95% confidence interval; *p < 0.05; **p < 0.01; ***p < 0.001.

## 4 Discussion

The impact on mental health during the pandemic context is indisputable. People have experienced fear, worry and stress as normal responses to uncertainty and crisis [Pan American Health Organization (PAHO), 2023]. However, compared to pre-pandemic conditions, confinement has worsened mental health. In fact, records from the Center for Disease Control and Prevention (CDC) in the United States showed that symptoms of anxiety and depression increased considerably during the year 2020, especially in at-risk populations such as young adults, women, racial/ethnic minorities, people with pre-existing illnesses, among others [National Center for Health Statistics (CDC), 2020]. On the same line, Pan American Health Organization (PAHO) (2023) estimates that in 2020, major depressive disorders increased by 35% and anxiety disorders by 32% in Latin America and the Caribbean.

At a descriptive level, the results of this study confirm the high prevalence of mental health issues during the pandemic among the population of young university students. The study identifies the presence of up to three critical frames and the prevalence of symptomatology in the sample. In this sense, the impact of the pandemic on the mental health of young people is worrying, given that around the world, they present a high prevalence of mental health problems, among which anxiety, stress, and depression symptoms stand out (Cao et al., 2020; Debowska et al., 2020; Wang et al., 2020; Wathelet et al., 2020; Li et al., 2021). This is understandable considering that, in addition to university closures that disrupted daily routines, learning, and socialization, young people faced fear of illness, loss of loved ones, increased adversity in their home environments, and digital exclusion [Sahu, 2020; Xie et al., 2020; Tasso et al., 2021; Wang et al., 2021; Organización Panamericana de la Salud (OPS), 2021b].

Regarding the gender differences found in the present study, where women showed greater difficulties in their mental health than male students, it should be considered that gender norms, values, and discrimination not only expose vulnerable people to mental health risks, but also impact in their help-seeking behavior, access to services, the response they receive from the health sector, and ultimately their mental health outcomes [Pan American Health Organization (PAHO), 2023]. The physical, mental and emotional burden faced by women in the COVID-19 pandemic crisis was related to having to assume responsibility for safeguarding their health while caring for household members, increased risk of violence, burnout from prolonged confinement, fear of contagion, and work as well as economic stress [Comisión Económica para América Latina (CEPAL), 2020]. This added load on women shows inequalities based on machismo, a phenomenon present in Peruvian and Latin-American societies [Amerio et al., 2021; Borrescio-Higa and Valenzuela, 2021; Dal Santo et al., 2022; Pan American Health Organization (PAHO), 2023].

In terms of the general objective of the study, positive and moderate relationships were found between resilience, social support, self-efficacy, and digital inclusion with mental health. In particular, general distress was found to be inversely related to resilience, social support, and academic self-efficacy. These findings reinforce the importance of working on coping strategies to deal with distress (Ampuero et al., 2022; Andersson et al., 2022; Noh and Park, 2022; Ortiz-Calvo et al., 2022); considering that resilience presents an inverse relationship with general distress and a direct relationship with psychological well-being (Beckstein et al., 2022; Calle and Urigüen, 2022; Nuñez and Vásquez, 2022; Valero et al., 2022; Xiao et al., 2023). This is complemented by the study of Li et al. (2021), who found that in the first weeks of confinement resilience functioned as a protective factor for mental health and that, when resilience is studied as a trait, social support serves as a buffer factor for the impact of low levels of resilience on mental health.

The results of this study show that in a context of crisis and social isolation, social support has played an important role for people and has shown to be one of the most powerful determinants of health. In this sense, it helps to recognize and use various social support resources to protect themselves from general or emotional distress (Wu et al., 2022), reducing mental health problems (Rueger et al., 2016; Uchino et al., 2016; Hu et al., 2020). Similarly, Dong et al. (2022) found that mental health is significantly related to perceived social support and life satisfaction; although after adjusting the control variables they found that perceived social support partially played a medium effect on mental health and life satisfaction.

As for academic self-efficacy, the findings confirm that there is indeed an inverse relationship with general distress, which is consistent with previous evidence (Alemany-Arrebola et al., 2020; Ampuero et al., 2022; Andersson et al., 2022; Carranza et al., 2022). A previous study conducted in Peru by Ampuero et al. (2022) found that one third of university students surveyed during confinement presented severe to extremely severe symptoms of depression, anxiety and stress and that academic self-efficacy worked as a protective factor with a moderate effect, as it allowed university students to be aware and confident of their own skills and abilities in different academic activities.

Additionally, a direct relationship was found between mental health and digital inclusion. It is understandable that, in the context of the pandemic and as a result of new distance education modalities, university students who did not have access to quality internet experienced higher emotional distress (Fawaz and Samaha, 2021). Although there is limited literature on the subject, research on groups that are digitally excluded highlights that they have more negative emotions (Shpigelman et al., 2021) and, when they do not have access to a computer, they have worse mental health recovery due to social isolation (Metherell et al., 2021).

Although digital inclusion had a heavier weight than academic self-efficacy in the Path Analysis model, it should be considered that this variable could function as a mediator rather than a predictor. In that sense, it was proposed that digital inclusion would have a mediating effect between resilience, social support, academic self-efficacy and general distress. In this way, it was found that it accomplishes a partial mediation with a low but significant effect size. This result implies that during the pandemic, the quality of the internet that the surveyed college students had partially mitigated the impact of social support, academic self-efficacy and resilience on the mental health of college students.

Social isolation during the pandemic meant a change in the way social ties were built and strengthened. The daily practices of socialization soon entered the virtual world and this implied new challenges as it was a space that united and separated at the same time. For university students, it also meant that their personal lives and their preparation as future professionals were affected, facing great challenges of adaptation, in many cases triggering increased stress, depression and anxiety (Maia and Dias, 2020; Leitón et al., 2023).

Therefore, it makes sense that the positive effect of social support on general distress may have been mitigated during the pandemic, when the quality of the internet was inadequate. This is because, in that context, the main form of social connection was through virtual means, which may have affected the effectiveness of social support in reducing general distress. Moreover, previous studies highlight that university students report that both the decrease in interaction with their teachers and the yearning for sharing with peers represented for them one of the main losses in the virtual education context (Coman et al., 2020; Tejedor et al., 2020; Zhou and Zhang, 2021).

Similarly, when a student does not have internet quality, their perception of academic self-efficacy would change, influencing their ability to cope with general distress. What is certain is that good connectivity can make a difference for good student performance (Ali, 2020; Debeş, 2021; Chanto and Loáiciga, 2022), so a low connection speed that limits or does not allow quality remote education would influence students to perceive that they are not performing adequately and could seriously affect their educational trajectory (Chanto and Loáiciga, 2022).

Regarding resilience, it was found that the mediation of digital inclusion is not as large compared to the variables of social support and academic self-efficacy. By understanding resilience as an individual trait, it is consequent that it is not so easily modified when there are factors in the environment that may represent a challenge for the person. In this line, it has been found that people with high levels of resilience have adapted without major difficulties to digital tools, as this helps people to solve problems more easily and have a more positive response to the new demands of society (Hurtado, 2021). Therefore, the quality of the Internet, a representative variable of digital inclusion in university students, would have a small but significant mediating role between resilience and general distress, since the former itself represents the ability to overcome adversities, such as the digital gap.

This study has certain limitations, such as the low response rate of online questionnaires, which may be due to the fact that the research was carried out at a critical time of the pandemic. Another limitation was that the conditions required for the study depended on good connectivity, which may have influenced the representativeness of people who did not have a good internet connection. Similarly, the research focused on digital inclusion regarding the quality of internet connection since access to technology and electronic devices was a condition that the sample of this study had; however, those students who presented difficulties could be the least willing to participate in this study. In this line, future research is recommended to deepen in the evaluation of digital competencies, which would account for other important aspects to be considered in the measurement of digital inclusion. Likewise, although the present research was able to record data from students in the midst of adapting to the crisis during the pandemic, it is necessary to continue evaluating its impact on the well-being of students, especially when partial or total distance education modalities are necessary.

In conclusion, this research underscores the pressing need to further explore the challenges of online education. The persistent lack of Internet connectivity in some regions highlights the importance of achieving effective digital inclusion, as its impact goes beyond education and directly affects students’ well-being and their resources to cope with the demands of everyday life. As we move forward in this digital age, we must remain committed to finding solutions that enable everyone to access quality education and the opportunities it provides.

## 5 Conclusion

In sum, mandatory distance education implied a profound educational and technological change that has had a negative impact on the mental health of university students. Emotional exhaustion, lack of face-to-face contact with others and self-demand accompanied by fear and uncertainty in the face of the virus and disease, have had an impact on their well-being due to the abrupt change in young people’s lives.

The results show that resilience, social support and academic self-efficacy play a protective role in reducing the levels of emotional or general distress and sustaining the mental health of university students. However, the study shows that when there is a digital divide around the quality of the Internet, the impact of these factors is affected. Digital inclusion, therefore, occupies a central discussion area for thinking about a more inclusive education, but also for discussing the determinants of college students’ mental health during the pandemic.

## Data availability statement

The original contributions presented in the study are included in the article/supplementary material, further inquiries can be directed to the corresponding author.

## Ethics statement

Ethical approval was not required for the studies involving humans because the investigation was a request of the university’s rectorate to know the mental health situation of its students. However, the committee was consulted and the ethical care was reviewed with them. After this, Given the emergency situation, the study was carried out. The studies were conducted in accordance with the local legislation and institutional requirements. The participants provided their written informed consent to participate in this study.

## Author contributions

MC: Conceptualization, Data curation, Methodology, Project administration, Writing – original draft, Writing – review & editing. AE: Formal analysis, Investigation, Methodology, Writing – original draft, Writing – review & editing. CC: Conceptualization, Data curation, Investigation, Writing – original draft, Writing – review & editing.
